# From Waldenström’s macroglobulinemia to aggressive diffuse large B-cell lymphoma: a whole-exome analysis of abnormalities leading to transformation

**DOI:** 10.1038/bcj.2017.72

**Published:** 2017-08-25

**Authors:** C Jiménez, S Alonso-Álvarez, M Alcoceba, G R Ordóñez, M García-Álvarez, M I Prieto-Conde, M C Chillón, A Balanzategui, R Corral, L A Marín, N C Gutiérrez, N Puig, M E Sarasquete, M González, R García-Sanz

**Affiliations:** 1 Hematology Department, University Hospital of Salamanca and Research Biomedical Institute of Salamanca (IBSAL), Salamanca, Spain; 2 Hematology Department, Central University Hospital of Asturias, Oviedo, Spain; 3 Center for Biomedical Research in Network of Cancer (CIBERONC), Salamanca, Spain; 4 DREAMgenics, Oviedo, Spain

## Abstract

Transformation of Waldenström’s macroglobulinemia (WM) to diffuse large B-cell lymphoma (DLBCL) occurs in up to 10% of patients and is associated with an adverse outcome. Here we performed the first whole-exome sequencing study of WM patients who evolved to DLBCL and report the genetic alterations that may drive this process. Our results demonstrate that transformation depends on the frequency and specificity of acquired variants, rather than on the duration of its evolution. We did not find a common pattern of mutations at diagnosis or transformation; however, there were certain abnormalities that were present in a high proportion of clonal tumor cells and conserved during this transition, suggesting that they have a key role as early drivers. In addition, recurrent mutations gained in some genes at transformation (for example, *PIM1*, *FRYL* and *HNF1B*) represent cooperating events in the selection of the clones responsible for disease progression. Detailed comparison reveals the gene abnormalities at diagnosis and transformation to be consistent with a branching model of evolution. Finally, the frequent mutation observed in the *CD79B* gene in this specific subset of patients implies that it is a potential biomarker predicting transformation in WM.

## Introduction

Waldenström’s macroglobulinemia (WM) is a neoplastic disease characterized by bone marrow infiltration with lymphoplasmacytic lymphoma and the presence of an IgM monoclonal component.^1^ Most patients show an indolent clinical course, and survival outcome has improved in recent years.^2^ However, this long-term evolution has led to an inherent increased risk of developing other malignancies including acute leukemia^3^ or non-Hodgkin lymphoma.^4^ Transformation into more aggressive histologies, in this case to diffuse large B-cell lymphoma (DLBCL), has been reported in up to 10% of WM patients.^5, 6^ Moreover, the prognosis of these patients appears to be worse than that for patients with *de novo* DLBCL and survival from the time of transformation is usually poor (median survival of ~2 years).^6, 7, 8^

The biological process of transformation in follicular lymphoma has been thoroughly studied.^9, 10, 11, 12, 13, 14, 15^ However, data from other indolent B-cell lymphoproliferative disorders are limited. No causes of WM transformation have yet been described, so understanding this process would be of great interest and would facilitate the development of new therapeutic strategies for improving the outcome of these patients. Recent advances in determining the WM mutational profile have revealed the presence of recurrent mutations, such as those of *MYD88*, *CXCR4* and *ARID1A*.^16, 17^ However, it is not known whether they are part of the mechanisms underlying the transformation to aggressive lymphoma. This event is of particular interest, especially when it involves such genetically distinct entities in appearance. Accordingly, WM seems to be a very homogeneous disease with a monotonous recurrent driver mutation (*MYD88* L265P),^18, 19^ and infrequent secondary alterations.^20^ Conversely, DLBCL harbors a wide range of diverse somatic mutations and genetic lesions that affect numerous intracellular pathways, thus making it intrinsically much more complex.^21, 22, 23^ However, the two entities share some alterations, such as *MYD88* L265P (observed in ~90% of WM and in 29% of ABC-type DLBCL),^18, 19, 21, 24^ *CD79A*/*CD79B* (15% WM and 10–15% DLBCL),^17, 20, 21, 23, 25, 26^ or copy number variations affecting 6q (40–60% WM and 20–40% DLBCL).^27, 28, 29, 30^

Transformation to DLBCL can occur at any time during the course of WM: at diagnosis, before treatment, during therapy and even 20 years after the initial diagnosis.^6^ In addition, no indicative clinicopathological feature or risk factor for developing DLBCL has so far been identified. No genomic studies of transformed WM patients have been carried out, so nothing is known about the biological signals that might explain particular susceptibilities. For this reason, the identification of genetic changes driving transformation is essential for a comprehensive understanding of the transition from WM to DLBCL that could help in the development of new diagnostic strategies and targeted therapies.

Against this background, we decided to perform the first whole-exome sequencing study focused on WM patients who experienced histological transformation. Integrating our new findings into our existing knowledge about the transformation of indolent lymphoproliferative syndromes to aggressive diseases should enable a transformational biological model to be derived, help define the molecular risk criteria for monitoring the course of the disease, and develop new preventive strategies. Our results revealed a higher incidence of mutations in the *CD79B* gene than in non-transformed WM patients, which could be interpreted as being a potential mechanism contributing to transformation. Finally, the comparison between diagnosis and transformation allowed us to establish an evolutionary pattern associated with the transforming event.

## Subjects and methods

### Subjects

Four patients diagnosed with transformed WM were included in the study. In three of them (patients 1, 2 and 4), matched tumor samples from diagnosis and transformation to DLBCL, as well as germline DNA were available for comparative study. One extra sample from patient 2 (corresponding to an event of WM progression without transformation) was also included. For patient 3, only DNA from germinal and transformed tumor cells was available. Cases were diagnosed using standard WHO classification criteria,^31^ including the new concepts that appear in the most recent review.^32^ The study and all procedures were performed in accordance with the Helsinki Declaration and were reviewed and approved by the Institutional Review Board of the Research Biomedical Institute of Salamanca. Informed consent was obtained from all patients.

The study group included one woman and three men, with a median age of 76 years (range, 62–82 years). The median time from diagnosis of WM to histological transformation was 52 months (range, 42–153 months). As an uncommon fact in this short series, we have to remark that two of the four patients had received treatment prior to histological transformation, and the other two (3 and 4) were chemo-naive. The complete patient characteristics are shown in Table 1.

### DNA extraction and quality assessment

The sample origins were as follows: (1) for WM tumor cells: bone marrow at diagnosis in all patients; (2) for DLBCL cells: spleen (patient 1), inguinal lymph node (patient 2) and bone marrow (patients 3 and 4) at transformation; and (3) for germline cells: peripheral blood samples in which no infiltration (<0.1%) was demonstrated by flow cytometry.

DNA was extracted by conventional methods: manually with the DNAzol reagent (MRC, Cincinnati, OH, USA) or automatically with the Maxwell system (Promega Corporation, Madison, WI, USA). Quantification and quality control of DNA were evaluated before enrichment and library preparation. DNA concentrations were measured using the Qubit fluorometer (Thermo Fisher Scientific, Inc., Waltham, MA, USA). DNA sample quality was assessed by gel electrophoresis. At least 1 μg of DNA was required for library preparations.

### Flow cytometry studies

Immunophenotypic evaluation was done by conventional methods, using panels of monoclonal antibodies previously described by our group^33^ and following the general recommendations of the EuroFlow group for the immunophenotypic evaluation of hematological malignancies.^34^

### Fluorescence *in situ* hybridization studies

Simple interphase fluorescence *in situ* hybridization was performed on cell nuclei from whole bone marrow samples using our previously published techniques.^35^ Del(6q), *MYC* and *BCL6* translocations t(14;18) (q21; q32) and del(17p) were analyzed with the probes Vysis CEP 6, LSI MYC Dual Color Break Apart Rearrangement (8q24), LSI BCL6 Dual Color Break Apart Rearrangement (3q27), Vysis IGH/BCL2 Dual Color Dual Fusion Translocation Probe t(14;18)(q32;q21) and LSI TP53 Probe (Abbott Molecular, Des Plaines, IL, USA), respectively. At least 100 cells were analyzed in all patient samples, applying Vysis scoring criteria. The cutoff point for the identification of alteration was set at ⩾10% cells with abnormal signal.

### V(D)J clonal rearrangements

V(D)J clonal rearrangements of WM and DLBCL matched samples were amplified and sequenced as described by the BIOMED-2/Euroclonality strategy.^36^

### Whole-exome sequencing

All diagnostic and transformation samples, as well as those for normal DNA matching, were sent for library construction and whole-exome sequencing at Macrogen, Inc. (Seoul, South Korea). Enrichment and generation of libraries were performed with SureSelectXT2 Human All Exon V5 of 51 MB (Agilent Technologies, Santa Clara, CA, USA) that uses cRNA probes of 120 nt. Paired-end sequencing was carried out in the Illumina HiSeq 2000 platform (Illumina, Inc., San Diego, CA, USA). The number of reads was set up according to each sample tumor infiltration defined by flow cytometry. Germinal samples were sequenced with a mean depth of 100 × and tumor DNA with a depth of 150 × or 200 ×, depending on whether the infiltration was greater or less than 40%, respectively.

### Sequencing data processing and bioinformatic analysis

DreamGenics (Oviedo, Spain) supervised the pre-processing and performed the initial bioinformatics analysis using algorithms and non-commercial pipelines to call variants, analyze and compare them. Briefly, data generated by the sequencer were converted to FastQ with the Illumina Consensus Assessment of Sequence and Variation version 1.8 software (https://support.illumina.com/sequencing/sequencing_software/bcl2fastq-conversion-software.html) and aligned to the human reference genome (Genome Reference Consortium human build 37, human genome 19) with BWA software.^37^ Variants were called using Atlas-SNP and Atlas-indel,^38^ discarding those with suboptimal quality indexes according to the pipeline criteria.

### Interpretation of variants

Only non-synonymous protein-coding alterations were considered. Criteria for filtering out single nucleotide polymorphisms were a frequency lower than 2% in the germinal sample and a population allele frequency <1% (according to the Human Gene Mutation Database).^39^ Some mutations were excluded on the basis of their distance with respect to the Agilent V5 probes. All samples were analyzed in pairs, with their corresponding germinal sample taken as reference.

Variant allele frequencies (VAFs) were corrected according to their tumor content defined by flow cytometry, in order to better estimate the percentage of tumor cells affected by each mutation. Variants with a corrected VAF of <10% were not included in the analysis.

### Enrichment analysis

Gene-set enrichment analysis was performed with the WEB-based GEne SeT AnaLysis Toolkit (WebGestalt) (http://www.webgestalt.org/webgestalt_2013/) to highlight categories and pathways present in the Gene Ontology and Pathway Commons databases. The statistical method employed was the hypergeometric test, with *P*-values adjusted by the Benjamini and Hochberg (1995) method.^40^

## Results

### Global non-synonymous variations

Overall, we found 421 non-synonymous variations (NSVs) at diagnosis and transformation in the four patients, distributed among 355 genes. Of these, only 39 were mutated exclusively in WM (at diagnosis, *n*=29; on progression, *n*=10) and 49 were present at the time of both events (diagnosis and transformation). All other genes (*n*=267) were mutated only in the DLBCL samples.

### Variant allelic frequency and percentage of tumor cells

**Figure 1:**
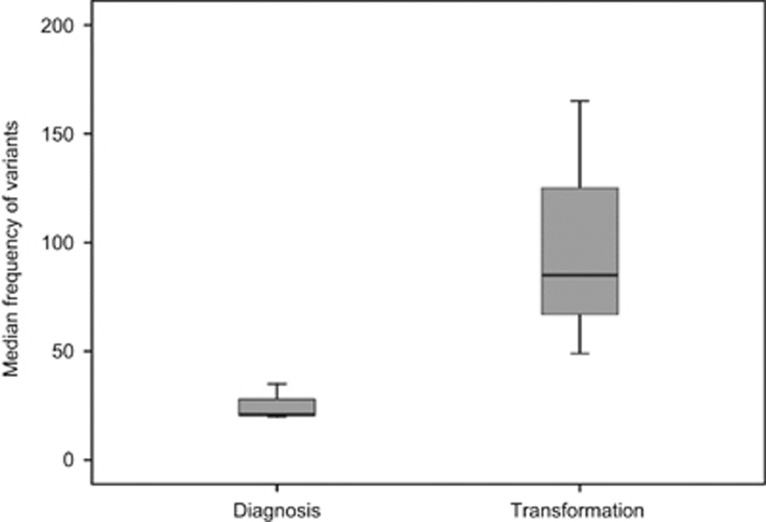
Frequency of mutations at diagnosis (WM) and transformation (DLBCL). Comparison of the median number of mutations of the four patients at diagnosis (*n*=21) and upon transformation (*n*=85).

**Figure 2:**
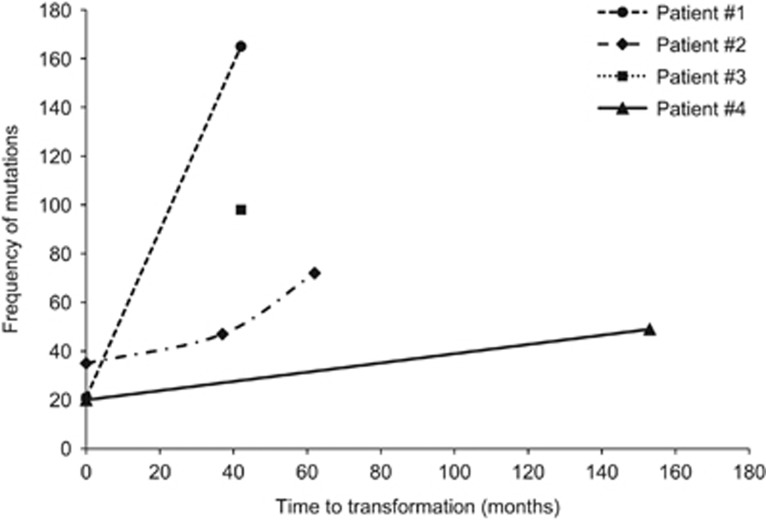
Representation of the total number of alterations at each moment (diagnosis, progression and transformation) versus the time to transformation. Patients 1 and 3 presented the fastest transformation (~3 years) and the highest frequency of variants (165 and 98, respectively). Patient 2 transformed in 5 years and had 72 mutations. Patient 4 took 13 years to transform and acquired only 49 alterations.

To understand the possible mechanisms leading to the transformation event, we compared the results in tumor cells of the two events studied. First, we observed a much higher frequency of mutated genes at transformation (median 85, range 49–165) than at diagnosis (median 21, range 20–35) (Figure 1). Accordingly, there was a median gain of 70 variants (range 29–144) per case during the transition from WM to DLBCL. Interestingly, the number of gains was not closely correlated with the interval between diagnosis and transformation (*R*^2^=0.51). However, we noticed that patients 1 and 3, who presented the fastest transformation (~3 years), contained more variants (165 and 98, respectively) than did patient 2, who transformed in 5 years and had 72 mutations, and patient 4, who exhibited the smoothest transformation, lasting 13 years, and acquired only 49 alterations (Figure 2).

### NSV present at diagnosis and transformation

**Figure 3:**
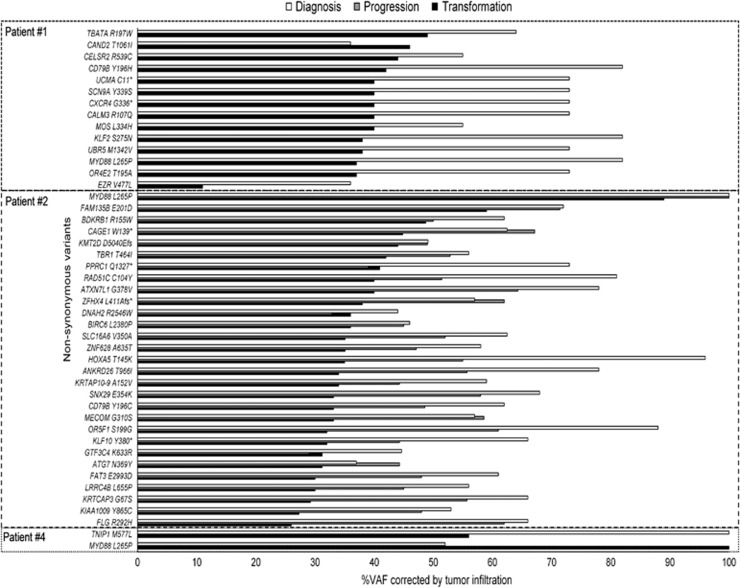
VAF of common mutations at diagnosis, progression and transformation for each patient. The percentage of tumor cells affected by a mutation decreased from diagnosis to transformation in most of the cases.

We found that the VAF was increased at transformation for almost all these common mutations, but this was attributed to the higher tumor infiltration present in the DLBCL compared with the corresponding WM sample (1, 40 vs 12% 2, 51.3 vs 22.4% 4, 54.6 vs 11%). Thus, the corrected VAF using flow cytometry showed the opposite pattern and VAFs were greater at diagnosis than at transformation (Figure 3). This means that, although there were more alterations at transformation, the percentage of tumor cells affected by each alteration was usually lower. This was confirmed using the globally corrected mean VAF for all NSVs present at diagnosis and at transformation (Table 2).

Not all patients had the same number of potential early drivers. Patients 2 and 4 had NSVs in 29 and 14 genes in common at diagnosis and transformation, respectively. However, patient 1 only shared two variants at the time of both events: *MYD88* and *TNIP1* (the latter having a higher VAF at diagnosis). This gene codes for a TNFAIP3-interacting protein that plays a role in regulating nuclear factor-κB activation, which is a frequent mechanism of alteration of cell cycle control in all lymphoid neoplasias.

### Recurrent NSV among different patients

*MYD88* L265P was present in all patients at both times (3/3 at diagnosis and 4/4 at transformation). The second most frequent was *CD79B* Y196C/H, which was found in 3/4 patients at transformation (75%) and in 2/3 at diagnosis (67%). VAFs of *CD79B* mutations were half of the *MYD88* in two patients and the same as *MYD88* in one patient, and kept that relation at baseline and transformation. *CELSR2*, *FAM135B*, *IGFN1* and *ZFHX4* also had recurrent variations (in at least two patients).

We then considered variants that could be detected at the time of only one of the events, that is, exclusively at diagnosis, progression or transformation (Supplementary Table I). Variations exclusively detected at diagnosis were not present in the lymphoma clone, implying that it does not provide any evolutionary advantage (passenger mutations). In contrast, recurrent variations exclusively present at the transformation stage (or that appeared late in the Waldenström’s clone) may be considered as temporally intermediate or late drivers that confer some advantage to the tumor clone. Five genes had recurrent NSVs acquired at the transformation stage: *FRYL* (MLL fusion partner in lymphoid leukemia), *HNF1B* (transcription factor whose expression is altered in some cancers), *PER3* (checkpoint protein that plays an important role in checkpoint activation, cell proliferation and apoptosis), *PIM1* (proto-oncogene with serine/threonine kinase activity involved in the pathogenesis of lymphoma) and *PTPRD* (tumor suppressor that contributes to the development of multiple cancers). All these data analyzed without inclusion of patient 3 are included in Supplementary Table II.

At this point, we carried out gene-set enrichment analysis of the genes modified at transformation (*n*=314) to search for cellular processes and the pathways affected. Genes were classified into the following functional categories: histone modification (*KMT2D*, *HDAC9*), chromatin modification (*PAX5*, *UBR5*), cell–cell adhesion (*CELSR2*, *TNIP1*), cell development (*WT1*, *CXCR4*), cell differentiation (*KIT*, *EZR*), protein modification (*KMD5C*, *KMD1B*), chromatin organization (*ARID1A*, *SOX1*), protein autophosphorylation (*PIM1*, *PRKD1*), chromosome organization (*HIST1H2BC*, *TP53*), transcription regulation (*RARB*, *PRDM1*) and protein kinase activity (*BCR*, *TGFA*). Focusing on pathways, those most significantly affected were: insulin growth factor-1 pathway (*CD79B*, *PPM1D*), interleukin-3-mediated signaling events (*TGFBRAP1*, *IFNA14*), insulin pathway (*COPA*, *CAD*), vascular endothelial growth factor and vascular endothelial growth factor receptor signaling network (*ACTN1*, *PELP1*), and nectin adhesion pathway (*ROBO1*, *NOS3*).

### Patterns of evolution from WM to DLBCL

As mentioned above, all patients shared some NSVs at both stages, but some others were exclusively detected at diagnosis or transformation. Accordingly, certain subclones present at diagnosis would have evolved by acquiring new mutations responsible for the transformation event (or the progression), whereas others would have been reduced (disappearing or becoming undetectable). Therefore, and despite both tumors (WM and DLBCL) having the same V(D)J rearrangement and an identical CDR3 region, suggesting a common progenitor cell, their evolution is consistent with a branching model. These findings should be considered with caution because of the low level of infiltration of diagnostic samples, which may lead to the underdetection of variants that are present at a low level in the tumor population.

**Figure 4:**
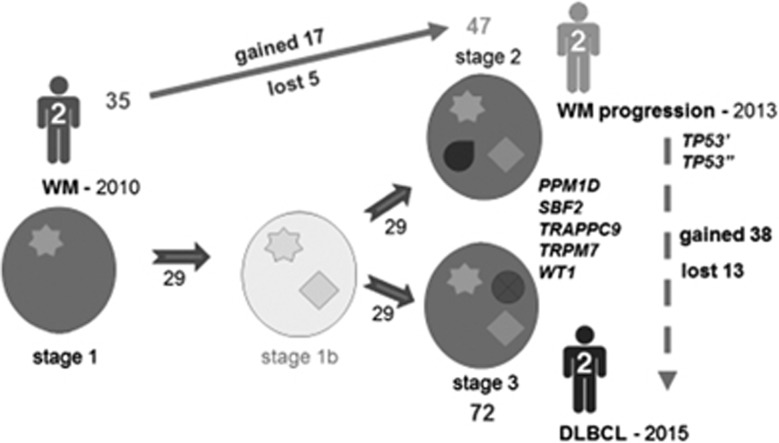
Evolution of WM in patient 2. This patient was diagnosed with WM in 2010 and transformed to DLBCL in 2015, with a symptomatic progression in 2013 before the transformation. We observed 35 mutations at diagnosis, 47 at relapse and 72 at transformation, including 29 alterations that were conserved at the times of the three events. The *PPM1D*, *SBF2*, *TRAPPC9*, *TRPM7* and *WT1* genes were mutated either at progression or transformation. By contrast, the two mutations found in *TP53* were seen at relapse but were lost by the time of transformation. This implies that the transformed final clone did not evolve from the same subclone as was responsible for progression, but from a previous one that would not yet have acquired the *TP53* mutations (among others).

This nonlinear evolutionary pattern was well illustrated by patient 2, who had a symptomatic progression of WM before transformation, and was studied at the time of all three events (Figure 4). During the evolution of his condition, we found mutations that were conserved from diagnosis to transformation (*n*=29). However, many other novel alterations not present at diagnosis appeared at progression (*n*=17) and transformation (*n*=38). Almost all of these were different, with five exceptions: *PPM1D*, *SBF2*, *TRAPPC9*, *TRPM7* and *WT1*. In the same way, some mutations present at diagnosis or acquired at progression (that is, two *TP53* mutations) disappeared or became undetectable (at the level of sensitivity used) by the time of disease transformation. This would mean that the transformed final clone did not come from the intermediate subclone responsible for progression, but from a previous minor subclone that only grew after progression.

## Discussion

WM patients may eventually experience histological transformation to DLBCL (2.4% transformation rate at 10 years), being at risk of poor outcome and short survival.^6, 7^ Understanding the biology and mechanisms underlying this process is important for identifying susceptible patients and for developing therapeutic strategies aimed at cancer control. In this study, we have carried out for the first time a whole-exome sequencing study of four cases with paired WM and transformed DLBCL samples in order to evaluate the genetic basis of this transition and to find genomic alterations and pathways that could be therapeutically targeted.

Maybe due to the small sample size, we could not find a unique genetic event responsible for WM transformation to DLBCL. In fact, our findings revealed extensive genetic heterogeneity with a large number of aberrations affecting many genes and pathways, reflecting the complexity associated with the transformation process. Even asymptomatic and chemotherapy-naive patients, such as patients 3 and 4, may develop an aggressive disease after more than 12 years (in the case of patient 4) with an untreated indolent WM. Many more alterations were associated with aggressive lymphoma development, although the number was inversely related to the time to transformation. Thus, patients showing the fastest transition (1 and 3) presented the greatest number of mutations. This could be explained by the onset of new mutations conferring a proliferative advantage on the harboring cell, leading to stronger competition during the evolution of the cancer.^41, 42, 43^

However, not all the events would be of equal importance in the pathogenesis of the disease. Mutations present at both events are likely to be spread in nearly all clones and to remain stable over time. In our WM cases, these variants had a higher VAF, so targeting these presumed early genetic events could lead to the elimination of all oncogenic clones. An example of these mutations could be the *MYD88* L265P, the only one present in all patients and at all times, a mutation with a well established role in both WM and DLBCL.^16, 24, 44^ However, as it is encountered in over 90% of WM cases at diagnosis, and only a small proportion of them will transform, no inference can be made about its role in transformation. In patient 1, it was the only alteration, together with *TNIP1*, recognized at both times. *TNIP1* is an essential gene for nuclear factor-κB activation, same as *MYD88*, which could highlight the need of more than one alteration in the nuclear factor-κB pathway to be significant for the transformation process, where nuclear factor-κB signaling is known to be involved.^12^ Furthermore, this patient also showed other differences with respect to the other cases, such as a much higher frequency of mutations at transformation (*n*=165), diffuse spleen infiltration by aggressive lymphoma cells, and an unmutated *CD79B*.

Now considering *CD79B*, this B-cell receptor-associated gene was frequently mutated, appearing in 2/3 cases (67%) at diagnosis and in 3/4 cases (75%) at transformation. VAFs of this mutation in relation to *MYD88* indicated that it was present in half of the tumor clone in two patients and in the whole clone in the other patient, staying the same at both moments. This variant affects the first tyrosine ITAM kinase domain of the receptor and has been described in 12% of ABC-type DLBCLs,^45^ in connection with the acquisition of the lymphoma phenotype. However, a limited oncogenic potential has been associated with *CD79B* mutations, since other alterations must occur in order to facilitate the transition to the aggressive lymphoma.^46, 47, 48^ In conventional WM, *CD79B* has been found to be mutated in ~10%,^17, 20, 25, 26^ so the high frequency reported here (3/4 of cases) is intriguing. Accordingly, we suggest that *CD79B* mutations identify a subgroup of WM with aggressive clinical evolution and a high risk of transformation.

With the exception of *MYD88* and *CD79B*, we observed a notable diversity in the mutational spectrum across samples, with few recurrent genes. Only *CELSR2* (growth factor), *FAM135B*, *IGFN1* and *ZFHX4* (transcription factor) were present in more than one patient. This recurrence should be taken into account, since another commonly mutated gene in WM (30% patients), *CXCR4*,^17, 20^ appeared in just one patient. Regardless of their incidence, relevant mutations seem to be present in the initial WM cell that will transform, suggesting that they may be involved in tumor initiation. Conversely, genes exclusively involved in transformation may represent cooperating events that interact with these pathogenic mutations. The most frequently altered genes were *FRYL*, *HNF1B*, *PER3*, *PIM1* and *PTPRD* (50% of patients). *PIM1* could be targeted with the PIM kinase inhibitors that have already shown activity in myeloma and acute myeloid leukemia.^49, 50^ However, some of the non-recurrent alterations could have been randomly acquired and may not be causally related to the pathogenesis of the disease.

Aberrations acquired at transformation probably cooperate with early initiating events in the selection of tumor clones responsible for disease progression. Nonetheless, considering the diversity of the alterations, many of them will not confer any advantage on the malignant cell. An illustrative case could be the presence of mutations in *TP53*. Deletions and mutations of this gene have been found to be related to poor prognosis in chronic lymphocytic leukemia,^51^ multiple myeloma^52^ and probably in WM.^53^ However, in our cases this gene appeared mutated at transformation in patient 1 and at progression in patient 2. Interestingly, this abnormality disappeared at transformation in the latter case, suggesting the loss of any advantage. Therefore, it is not easy to determine what prompts aggressive behavior: the genes, the number of alterations, the clone in which they arise, the pathway affected, the cell function deregulated or, most likely, a combination of all of these factors. These matters should be addressed in further studies with the ultimate aim of developing therapeutic strategies that may disrupt these mechanisms, thereby completely preventing transformation.

Finally, we observed that the transformation process seemed to be consistent with part of a branching model of evolution in which only clones containing driver mutations evolve to more aggressive populations by acquiring new aberrations. Identical scenarios have been reported in multiple myeloma,^54^ follicular lymphoma,^12^ chronic lymphocytic leukemia,^55^ acute myeloid leukemia,^42^ acute lymphoblastic leukemia^56^ and even solid tumors.^57^ Nevertheless, this needs to be confirmed in further analyses of single cells. Likewise, it would also be interesting to establish whether there is a progenitor tumor cell that is common to both diseases and that is responsible for their pathogenesis. In this study, the analysis of the V(D)J rearrangement and CDR3 region of the immunoglobulin heavy chain gene in the WM and their matched lymphoma samples, confirmed that they belonged to the same clone. The concept of the tumor-initiating cell is already established for leukemia^58^ and follicular lymphoma,^59, 60^ and it would be supported in our case by the presence from the outset of certain mutations that are shared by the entire tumor population, as they remain clonally stable throughout the entire course of the disease. DLBCL would then arise from these precursors, showing a higher (as in patients 2 and 4) or lower (patient 1) degree of genetic similarity to the WM clone.

In conclusion, although this is merely the first step and we were not able to identify a unique genetic event responsible for WM transformation to DLBCL, it appears that certain alterations may contribute to the onset of aggressive disease. Those genes frequently mutated at diagnosis in this subset of patients who suffer disease transformation compared to conventional WM (*CD79B*) may be considered as potential biomarkers for predicting the risk of transformation in prospective studies. Additional research is needed to better understand the biology of this process and to facilitate the design of preventive therapies targeted during the early evolutionary pathways that are responsible for transformation. This knowledge will enable us to improve the outcome of these patients.

## Figures and Tables

**Table 1:** Clinical characteristics of patients

*Patient*	*1*	*2*	*3*	*4*
*WM*				
Age at diagnosis	62 (2009)	82 (2010)	81 (2003)	72 (2002)
Clinical symptoms				
Anemia	Yes	Yes	No	Yes
Polyadenopathies	Yes	No	No	No
LDH elevation	Yes	No	No	No
Hyperviscosity	No	No	Yes	No
Others	No	No	No	No
BM infiltration (FCM)	12%	22%	16%	11%
Frontline therapy (year)	R-VD (2009)	R-CD (2010)	None	W&W
Therapies at relapse (year)	FC (2011)	FC (2013)RB (2013/2014)R-VD (2015)	None	None
				
*Transformation to DLBCL*				
Time to transformation (years)	3	5	3	13
Clinical symptoms				
Anemia	Yes	No	Yes	Yes
Polyadenopathies	No	Yes	No	No
LDH elevation	Yes	Yes	Yes	Yes
B symptoms	Yes	No	Yes	Yes
Others	Splenomegaly			
Tumor infiltration (FCM)	40% (Spleen)	50% (Adenopathy)	16% (BM)^a^	43% (BM)
DLBCL therapy	None	GemOx (2015) Ibrutinib (2016)	None	RCOP (2015) (reduced)
Status	Dead	Palliative care	Dead	Dead

Abbreviations: BM, bone marrow; DLBCL, diffuse large B-cell lymphoma; FC, fludarabine, cyclophosphamide; FCM, flow cytometry; GemOx, gemcitabine, oxaliplatin; LDH, lactate dehydrogenase; RB, rituximab, bendamustine; R-CD, rituximab, cyclophosphamide, dexamethasone; RCOP, rituximab, cyclophosphamide, vincristine, prednisone; R-VD, rituximab, bortezomid, dexamethasone; WM, Waldeström macroglobulinemia; W&W, watch and wait (observation).

a BM: 8% WM and 8% DLBCL.

**Table 2:** Mean VAF of common and exclusive mutations at diagnosis and transformation

	*Patient 1*		*Patient 2*		*Patient 4*	
	*Diagnosis*	*Transformation*	*Diagnosis*	*Transformation*	*Diagnosis*	*Transformation*
Mean VAF of common mutations	76%	78%	64%	37%	66%	38%
Mean VAF of exclusive mutations	68%	51%	30%	22%	45%	16%

Abbreviation: VAF, variant allele frequency.

## Supplementary Material

Supplementary Table I

Supplementary Table II
