# Supplementary material for: From Waldenström’s macroglobulinemia to aggressive diffuse large B-cell lymphoma: a whole-exome analysis of abnormalities leading to transformation
Source: Blood Cancer J. 2017 Aug 25;7(8):e591–. doi: 10.1038/bcj.2017.72 (PMC5596383; doi:10.1038/bcj.2017.72)
Supplement: Supplementary Table I [file bcj201772x1.docx]

**Supplemental Table I.- List of mutations present in each patient at one-time moment.**

| Gen | Mutation | % VAF | %VAF corrected by tumor infiltration | Patient # | Time point |
| --- | --- | --- | --- | --- | --- |
| *ABCB4* | p.T1255K | 22 | 56 | 1 | Transformation |
| *ACTN1* | p.V873M | 12 | 31 | 1 | Transformation |
| *ADAMTS16* | p.A514E | 16 | 39 | 1 | Transformation |
| *ADAMTS2* | p.N949K | 22 | 55 | 1 | Transformation |
| *ADAMTS9* | p.L1420M | 12 | 24 | 2 | Transformation |
| *AGPAT4* | p.P295L | 11 | 21 | 2 | Transformation |
| *AIM1* | p.G638D | 21 | 52 | 1 | Transformation |
| *AJAP1* | p.P146L | 10 | 81 | 1 | Diagnosis |
| *AMER1* | p.V633F | 16 | 39 | 1 | Transformation |
| *ANKRD52* | p.T665A | 7 | 14 | 2 | Transformation |
| *AP3B2* | p.V231M | 9 | 12 | 2 | Progression |
| *ARID1A* | p.M872V | 17 | 43 | 1 | Transformation |
| *ASTN2* | p.G539V | 28 | 71 | 1 | Transformation |
| *ASXL3* | p.P1003S | 9 | 13 | 2 | Progression |
| *AVPR1B* | p.S220N | 8 | 68 | 1 | Diagnosis |
| *B3GNT6* | p.L316W | 9 | 80 | 4 | Diagnosis |
| *BCCIP* | p.L178R | 26 | 64 | 1 | Transformation |
| *BGN* | p.R183W | 8 | 68 | 1 | Diagnosis |
| *BHLHE22* | p.G217S | 7 | 67 | 4 | Diagnosis |
| *BTG1* | p.S43N | 13 | 26 | 2 | Transformation |
| *C10orf82* | p.R48G | 26 | 64 | 1 | Transformation |
| *C17orf107* | p.A144T | 26 | 64 | 1 | Transformation |
| *C19orf59* | p.M111I | 13 | 26 | 2 | Transformation |
| *C21orf49* | p.G68* | 3 | 28 | 4 | Diagnosis |
| *C6orf141* | p.R218* | 14 | 35 | 1 | Transformation |
| *CAD* | p.Y60C | 23 | 57 | 1 | Transformation |
| *CAPN13* | p.D541N | 18 | 44 | 1 | Transformation |
| *CASC5* | p.T2217I | 7 | 12 | 4 | Transformation |
| *CCDC88A* | p.K714E | 20 | 50 | 1 | Transformation |
| *CCNA1* | p.T220N | 19 | 49 | 1 | Transformation |
| *CDH7* | p.A591V | 14 | 34 | 1 | Transformation |
| *CDH9* | p.G250V | 23 | 56 | 1 | Transformation |
| *CEP170B* | p.A795T | 25 | 61 | 1 | Transformation |
| *CHST2* | p.L68F | 17 | 44 | 1 | Transformation |
| *CHST2* | p.L68V | 16 | 41 | 1 | Transformation |
| *CLPB* | p.A222T | 24 | 59 | 1 | Transformation |
| *CMYA5* | p.E3551* | 18 | 45 | 1 | Transformation |
| *CNTNAP2* | p.S53P | 19 | 47 | 1 | Transformation |
| *COPA* | p.D351H | 6 | 10 | 4 | Transformation |
| *CRISPLD1* | p.S102I | 18 | 44 | 1 | Transformation |
| *CRY2* | p.I521T | 4 | 36 | 4 | Diagnosis |
| *CRYGN* | p.R96C | 18 | 44 | 1 | Transformation |
| *CTNNA3* | p.L814* | 19 | 48 | 1 | Transformation |
| *CXorf65* | p.F2L | 22 | 55 | 1 | Transformation |
| *DCHS2* | p.G924R | 13 | 100 | 1 | Diagnosis |
| *DDX23* | p.R368K | 20 | 51 | 1 | Transformation |
| *DDX53* | p.P288S | 13 | 32 | 1 | Transformation |
| *DENND6B* | p.F94L | 22 | 54 | 1 | Transformation |
| *DLGAP2* | p.R237W | 23 | 58 | 1 | Transformation |
| *DNAH7* | p.L3066H | 9 | 18 | 2 | Transformation |
| *DRP2* | p.R410H | 16 | 29 | 4 | Transformation |
| *DSC3* | p.K653Q | 5 | 10 | 2 | Transformation |
| *DSCAML1* | p.V189I | 44 | 63 | 2 | Progression |
| *DSG2* | p.T108N | 8 | 11 | 2 | Progression |
| *EHF* | p.G14S | 8 | 14 | 4 | Transformation |
| *EIF4G3* | p.Q1558K | 11 | 90 | 1 | Diagnosis |
| *ENAH* | p.E217D | 4 | 35 | 1 | Diagnosis |
| *ENTPD2* | p.G340E | 19 | 48 | 1 | Transformation |
| *ERAP2* | p.H145L | 18 | 44 | 1 | Transformation |
| *ERBB3* | p.T32A | 21 | 53 | 1 | Transformation |
| *ERCC4* | p.T29I | 10 | 18 | 4 | Transformation |
| *ETAA1* | p.E25D | 29 | 71 | 1 | Transformation |
| *FAM135B* | p.N447K | 17 | 43 | 1 | Transformation |
| *FAM149A* | p.R364W | 20 | 100 | 1 | Diagnosis |
| *FAM154A* | p.G77W | 30 | 76 | 1 | Transformation |
| *FAM209A* | p.S6L | 35 | 88 | 1 | Transformation |
| *FAM83F* | p.F204Y | 8 | 15 | 2 | Transformation |
| *FAM84A* | p.R220C | 22 | 55 | 1 | Transformation |
| *FARP2* | p.V1003I | 23 | 57 | 1 | Transformation |
| *FAT4* | p.S4952F | 6 | 11 | 4 | Transformation |
| *FBN3* | p.I1504T | 21 | 52 | 1 | Transformation |
| *FEZF1* | p.P76T | 21 | 51 | 1 | Transformation |
| *FLJ00104* | p.H240Afs*21 | 17 | 41 | 1 | Transformation |
| *FLT4* | p.T70S | 26 | 37 | 2 | Progression |
| *FNBP4* | p.A900T | 26 | 66 | 1 | Transformation |
| *FREM1* | p.H1258Q | 12 | 23 | 2 | Transformation |
| *FRYL* | p.A355V | 16 | 41 | 1 | Transformation |
| *FRYL* | p.N2282K | 7 | 14 | 2 | Transformation |
| *FSD1* | p.R332C | 17 | 34 | 2 | Transformation |
| *GABRA3* | p.Q235L | 31 | 77 | 1 | Transformation |
| *GLIS3* | p.T322M | 6 | 12 | 4 | Transformation |
| *GLOD4* | p.F199S | 7 | 14 | 2 | Transformation |
| *GLRA1* | p.R57K | 13 | 33 | 1 | Transformation |
| *GOT2* | p.A213S | 8 | 16 | 2 | Transformation |
| *GPATCH8* | p.S469G | 10 | 84 | 1 | Diagnosis |
| *GPC5* | p.S245F | 17 | 42 | 1 | Transformation |
| *GPR116* | p.L1198P | 13 | 32 | 1 | Transformation |
| *GPR6* | p.L210P | 24 | 60 | 1 | Transformation |
| *GRIPAP1* | p.F10S | 32 | 79 | 1 | Transformation |
| *GRM3* | p.R68H | 16 | 39 | 1 | Transformation |
| *GRM6* | p.R731Q | 7 | 10 | 2 | Progression |
| *HDAC9* | p.R663Q | 19 | 48 | 1 | Transformation |
| *HIST1H2BC* | p.E72D | 20 | 49 | 1 | Transformation |
| *HIVEP1* | p.S2574T | 19 | 48 | 1 | Transformation |
| *HNF1B* | p.S115N | 22 | 54 | 1 | Transformation |
| *HNF1B* | p.Q147H | 16 | 32 | 2 | Transformation |
| *HOXA3* | p.E69D | 17 | 25 | 2 | Progression |
| *IGFN1* | p.T1549R | 8 | 34 | 2 | Diagnosis |
| *IGLL5* | p.S47R | 24 | 61 | 1 | Transformation |
| *IGSF1* | p.M335I | 19 | 48 | 1 | Transformation |
| *IMPA1* | p.I27M | 23 | 59 | 1 | Transformation |
| *IRF2BP2* | p.Y43D | 16 | 41 | 1 | Transformation |
| *ITIH2* | p.C261Y | 21 | 52 | 1 | Transformation |
| *JARID2* | p.D744N | 12 | 22 | 4 | Transformation |
| *JMJD6* | p.A283T | 6 | 11 | 4 | Transformation |
| *KALRN* | p.L1654F | 42 | 100 | 1 | Transformation |
| *KCNJ1* | p.F65I | 6 | 12 | 2 | Transformation |
| *KDM1B* | p.A720T | 19 | 46 | 1 | Transformation |
| *KDM3B* | p.I1683M | 19 | 48 | 1 | Transformation |
| *KDM4C* | p.W773* | 18 | 46 | 1 | Transformation |
| *KDM5C* | p.S169A | 41 | 100 | 1 | Transformation |
| *KHSRP* | p.C436R | 19 | 48 | 1 | Transformation |
| *KIAA0100* | p.Q687R | 9 | 74 | 1 | Diagnosis |
| *KIAA0319L* | p.L131* | 7 | 12 | 4 | Transformation |
| *KIT* | p.S465F | 21 | 52 | 1 | Transformation |
| *KLF17* | p.E30K | 10 | 19 | 2 | Transformation |
| *KMT2D* | p.L3154* | 13 | 25 | 2 | Transformation |
| *KRT4* | p.V133F | 20 | 49 | 1 | Transformation |
| *KRTAP4-2* | p.G16R | 8 | 16 | 2 | Transformation |
| *KTN1* | p.N970S | 14 | 28 | 2 | Transformation |
| *L1TD1* | p.K812E | 20 | 49 | 1 | Transformation |
| *LILRB4* | p.S146* | 8 | 14 | 4 | Transformation |
| *LIN54* | p.P311R | 6 | 12 | 2 | Transformation |
| *LPCAT1* | p.S500C | 7 | 14 | 4 | Transformation |
| *LPPR3* | p.L41V | 6 | 10 | 4 | Transformation |
| *LRP4* | p.P183S | 15 | 37 | 1 | Transformation |
| *MAGEE1* | p.R658* | 15 | 38 | 1 | Transformation |
| *MALRD1* | p.W180C | 20 | 49 | 1 | Transformation |
| *MCF2L2* | p.R120* | 10 | 83 | 1 | Diagnosis |
| *MCHR2* | p.F208Sfs*5 | 11 | 19 | 4 | Transformation |
| *MCM8* | p.T135P | 7 | 13 | 2 | Transformation |
| *MDN1* | p.R3986W | 5 | 10 | 4 | Transformation |
| *MED13* | p.P835R | 5 | 38 | 1 | Diagnosis |
| *MELK* | p.Y267D | 19 | 47 | 1 | Transformation |
| *MEP1A* | p.P676L | 20 | 50 | 1 | Transformation |
| *MLLT3* | p.T319Vfs*8 | 10 | 26 | 1 | Transformation |
| *MMAA* | p.G35R | 6 | 11 | 4 | Transformation |
| *MMP3* | p.R53K | 31 | 77 | 1 | Transformation |
| *MPEG1* | p.S273F | 17 | 43 | 1 | Transformation |
| *MT-ND5* | p.L266Pfs*3 | 34 | 62 | 4 | Transformation |
| *MUSK* | p.D725N | 21 | 52 | 1 | Transformation |
| *MYO18A* | p.S35R | 12 | 23 | 2 | Transformation |
| *MYO5A* | p.R178Q | 16 | 41 | 1 | Transformation |
| *NCKAP5* | p.E1307K | 7 | 13 | 4 | Transformation |
| *NCOA2* | p.P724S | 7 | 33 | 2 | Diagnosis |
| *NDST3* | p.I267T | 19 | 47 | 1 | Transformation |
| *NEK4* | p.R777K | 18 | 45 | 1 | Transformation |
| *NGRN* | p.A63G | 14 | 36 | 1 | Transformation |
| *NOS3* | p.R670W | 19 | 48 | 1 | Transformation |
| *NRBP1* | p.R104C | 20 | 50 | 1 | Transformation |
| *NRP1* | p.R334H | 10 | 84 | 1 | Diagnosis |
| *NSMF* | p.Q310K | 9 | 76 | 1 | Diagnosis |
| *NTSR1* | p.R372H | 21 | 53 | 1 | Transformation |
| *ODF3B* | p.S44P | 39 | 97 | 1 | Transformation |
| *ODF3B* | p.P33A | 19 | 49 | 1 | Transformation |
| *OR2T4* | p.P127H | 15 | 39 | 1 | Transformation |
| *OR4C6* | p.C95* | 14 | 27 | 2 | Transformation |
| *OR4K5* | p.I219S | 7 | 12 | 4 | Transformation |
| *OR4K5* | p.F211C | 6 | 11 | 4 | Transformation |
| *OR4N4* | p.N137T | 16 | 40 | 1 | Transformation |
| *OR5AS1* | p.Q193H | 7 | 12 | 4 | Transformation |
| *OR5M9* | p.L129F | 15 | 29 | 2 | Transformation |
| *OSBPL10* | p.G53V | 16 | 41 | 1 | Transformation |
| *PAPOLA* | p.Q17L | 7 | 12 | 4 | Transformation |
| *PATZ1* | p.K299Q | 18 | 46 | 1 | Transformation |
| *PAX5* | p.P190R | 6 | 12 | 2 | Transformation |
| *PCDH20* | p.I544M | 21 | 53 | 1 | Transformation |
| *PCDHA3* | p.V464M | 17 | 42 | 1 | Transformation |
| *PCDHGB3* | p.T406R | 5 | 10 | 4 | Transformation |
| *PCLO* | p.E2925D | 3 | 29 | 4 | Diagnosis |
| *PCLO* | p.R1613Q | 9 | 16 | 4 | Transformation |
| *PCNT* | p.R169H | 10 | 44 | 2 | Diagnosis |
| *PER3* | p.H509Y | 15 | 37 | 1 | Transformation |
| *PHB* | p.L241V | 16 | 40 | 1 | Transformation |
| *PIM1* | p.K24N | 34 | 85 | 1 | Transformation |
| *PIM1* | p.M1_M87del | 31 | 77 | 1 | Transformation |
| *PIM1* | p.L25V | 20 | 49 | 1 | Transformation |
| *PIM1* | p.L182V | 18 | 45 | 1 | Transformation |
| *PIM1* | p.L129F | 17 | 43 | 1 | Transformation |
| *PIM1* | p.F116V | 16 | 41 | 1 | Transformation |
| *PIM1* | p.Q37P | 16 | 41 | 1 | Transformation |
| *PIM1* | p.P125S | 16 | 40 | 1 | Transformation |
| *PIM1* | p.L184F | 14 | 35 | 1 | Transformation |
| *PIM1* | p.Q37* | 13 | 31 | 1 | Transformation |
| *PIM1* | p.G50D | 12 | 30 | 1 | Transformation |
| *PIM1* | p.P81S | 11 | 28 | 1 | Transformation |
| *PIM1* | p.G55D | 9 | 23 | 1 | Transformation |
| *PIM1* | p.S146N | 8 | 20 | 1 | Transformation |
| *PIM1* | p.E135* | 6 | 16 | 1 | Transformation |
| *PKHD1* | p.N1532* | 12 | 30 | 1 | Transformation |
| *PLEKHA7* | p.I157M | 31 | 77 | 1 | Transformation |
| *POLR2F* | p.M57K | 25 | 63 | 1 | Transformation |
| *POTEC* | p.K36R | 3 | 28 | 1 | Diagnosis |
| *PPL* | p.T564M | 16 | 31 | 2 | Transformation |
| *PRDM1* | p.A174P | 26 | 65 | 1 | Transformation |
| *PRDM6* | p.P317L | 22 | 56 | 1 | Transformation |
| *PRKD1* | p.R695W | 19 | 48 | 1 | Transformation |
| *PSG9* | p.*427W | 16 | 39 | 1 | Transformation |
| *PTPRD* | p.L673H | 25 | 63 | 1 | Transformation |
| *PTPRD* | p.R1323H | 9 | 17 | 4 | Transformation |
| *RARB* | p.F82L | 18 | 34 | 2 | Transformation |
| *RGS1* | p.T94I | 22 | 54 | 1 | Transformation |
| *RGS22* | p.R1108W | 23 | 57 | 1 | Transformation |
| *RNPC3* | p.Y49D | 36 | 89 | 1 | Transformation |
| *ROBO1* | p.P1529S | 15 | 37 | 1 | Transformation |
| *RYR3* | p.Q1493H | 6 | 12 | 2 | Transformation |
| *SCN7A* | p.F724L | 8 | 16 | 2 | Transformation |
| *SDK1* | p.Q874* | 28 | 69 | 1 | Transformation |
| *SEL1L2* | p.T639M | 20 | 50 | 1 | Transformation |
| *SEMA3G* | p.E186D | 6 | 46 | 1 | Diagnosis |
| *SEMA6D* | p.V758L | 20 | 50 | 1 | Transformation |
| *SETBP1* | p.Y571Hfs*32 | 14 | 34 | 1 | Transformation |
| *SGSM1* | p.L861R | 21 | 52 | 1 | Transformation |
| *SHANK2* | p.V80M | 23 | 59 | 1 | Transformation |
| *SHROOM3* | p.P982T | 13 | 33 | 1 | Transformation |
| *SI* | p.T543I | 6 | 47 | 1 | Diagnosis |
| *SIAH2* | p.A126V | 16 | 40 | 1 | Transformation |
| *SIK1* | p.R432P | 11 | 21 | 2 | Transformation |
| *SLC13A1* | p.A383V | 13 | 26 | 2 | Transformation |
| *SLC27A1* | p.R462C | 18 | 44 | 1 | Transformation |
| *SLC35F5* | p.R44* | 5 | 10 | 2 | Transformation |
| *SLC6A8* | p.A345S | 11 | 27 | 1 | Transformation |
| *SLC9A3* | p.L299R | 19 | 47 | 1 | Transformation |
| *SLITRK3* | p.F943I | 6 | 11 | 4 | Transformation |
| *SLITRK4* | p.S358I | 21 | 41 | 2 | Transformation |
| *SOX1* | p.Q17* | 22 | 56 | 1 | Transformation |
| *SPAG17* | p.H529Y | 16 | 41 | 1 | Transformation |
| *SPATA17* | p.A219T | 16 | 39 | 1 | Transformation |
| *SPEF2* | p.E116D | 15 | 37 | 1 | Transformation |
| *SPEG* | p.N1538D | 7 | 14 | 2 | Transformation |
| *SPHKAP* | p.C84F | 9 | 13 | 2 | Progression |
| *SPTBN5* | p.A452V | 25 | 61 | 1 | Transformation |
| *SRP54* | p.D138G | 14 | 34 | 1 | Transformation |
| *ST6GALNAC6* | p.R202Q | 9 | 17 | 4 | Transformation |
| *STARD9* | p.C3057Y | 7 | 12 | 4 | Transformation |
| *STEAP1* | p.E217* | 6 | 11 | 4 | Transformation |
| *SUN1* | p.A556V | 23 | 59 | 1 | Transformation |
| *SYCP2L* | p.L161Wfs*8 | 11 | 21 | 2 | Transformation |
| *TAS2R10* | p.A148V | 17 | 44 | 1 | Transformation |
| *TBCD* | p.P485S | 18 | 45 | 1 | Transformation |
| *TBL1XR1* | p.G247E | 22 | 32 | 2 | Progression |
| *TBX22* | p.G109R | 32 | 63 | 2 | Transformation |
| *TCEAL2* | p.Q15P | 6 | 15 | 1 | Transformation |
| *TCHH* | p.R953H | 8 | 15 | 4 | Transformation |
| *TDRD6* | p.E988* | 14 | 26 | 4 | Transformation |
| *TEX13A* | p.R77C | 11 | 95 | 1 | Diagnosis |
| *TEX29* | p.G122E | 8 | 11 | 2 | Progression |
| *TGFA* | p.A85V | 19 | 48 | 1 | Transformation |
| *TGFBRAP1* | p.G74S | 20 | 51 | 1 | Transformation |
| *TJP3* | p.G878E | 22 | 54 | 1 | Transformation |
| *TP53* | p.T211I | 26 | 65 | 1 | Transformation |
| *TP53* | p.R196* | 19 | 27 | 2 | Progression |
| *TP53* | p.R248W | 11 | 15 | 2 | Progression |
| *TRANK1* | p.K135N | 7 | 12 | 4 | Transformation |
| *TRIM62* | p.E52G | 24 | 60 | 1 | Transformation |
| *TRPM3* | p.R571H | 16 | 32 | 2 | Transformation |
| *TTC40* | p.D2439N | 23 | 57 | 1 | Transformation |
| *TTLL2* | p.S329P | 9 | 16 | 4 | Transformation |
| *TYRO3* | p.R490C | 21 | 52 | 1 | Transformation |
| *UBE2A* | p.F41S | 37 | 93 | 1 | Transformation |
| *UBR7* | p.D116G | 9 | 17 | 2 | Transformation |
| *UCMA* | p.C11* | 22 | 40 | 4 | Transformation |
| *UGT3A1* | p.M114T | 19 | 47 | 1 | Transformation |
| *UNC13B* | p.G355C | 35 | 87 | 1 | Transformation |
| *UNC13C* | p.S818G | 19 | 48 | 1 | Transformation |
| *UNC93B1* | p.N44T | 38 | 95 | 1 | Transformation |
| *VSIG10L* | p.A859P | 5 | 42 | 1 | Diagnosis |
| *VWF* | p.S1506L | 4 | 20 | 2 | Diagnosis |
| *WDR93* | p.L490M | 3 | 28 | 4 | Diagnosis |
| *ZC3H12B* | p.V764M | 34 | 84 | 1 | Transformation |
| *ZFHX4* | p.K3332R | 6 | 15 | 1 | Transformation |
| *ZMYM2* | p.A1258V | 7 | 57 | 1 | Diagnosis |
| *ZNF181* | p.H451P | 5 | 22 | 2 | Diagnosis |
| *ZNF324* | p.A226S | 8 | 15 | 4 | Transformation |
| *ZNF385D* | p.T385S | 17 | 42 | 1 | Transformation |
| *ZNF469* | p.R473W | 24 | 59 | 1 | Transformation |
| *ZNF565* | p.V296A | 23 | 56 | 1 | Transformation |
| *ZNF84* | p.I508S | 27 | 67 | 1 | Transformation |
