# Supplementary material for: From Waldenström’s macroglobulinemia to aggressive diffuse large B-cell lymphoma: a whole-exome analysis of abnormalities leading to transformation
Source: Blood Cancer J. 2017 Aug 25;7(8):e591–. doi: 10.1038/bcj.2017.72 (PMC5596383; doi:10.1038/bcj.2017.72)
Supplement: Supplementary Table II [file bcj201772x2.docx]

| Global non-synonymous variations | 326 |
| --- | --- |
| Genes affected | 300 |
| Genes mutated only in DLBCL | 212 |
| Median of mutated genes at transformation | 72 [49-165] |
| Alterations present at diagnosis and transformation |  |
| *MYD88* L265P | 100% (3/3) of patients |
| *CD79B* Y196H/C | 67% (2/3) of patients |
| Other recurrent alterations: *FAM135B* and *ZFHX4* | 33% at diagnosis and 67% at transformation |
| Recurrent genes at transformation | *FRYL*, *HNF1B* and *PTPRD* |

**Supplemental Table II.- Results excluding patient #3.**
